# Chest Pain and Electrocardiographic Changes in a Child With Duchenne Muscular Dystrophy

**DOI:** 10.7759/cureus.26105

**Published:** 2022-06-20

**Authors:** Hanan Salah Al Hajri, Eman Mahmoud El Husseiny, Hasan Qayyum

**Affiliations:** 1 Emergency Department, Shaikh Shakhbout Medical City, Abu Dhabi, ARE; 2 Emergency Department, Sheikh Khalifa Medical City, Abu Dhabi, ARE

**Keywords:** pediatric resuscitation, pediatric emergency department, ecg abnormalities, emergency md, duchenne muscular dystrophy (dmd)

## Abstract

A 12-year-old boy known to have Duchenne muscular dystrophy presented to our Emergency Department with acute onset central chest pain.

A 12-lead electrocardiogram (ECG) was performed showing ST-segment elevation with reciprocal changes. An echocardiogram showed reduced left ventricular systolic function with an ejection fraction of 45%. Initial cardiac biomarkers were significantly elevated, with troponin-T result recorded at 7,065 ng/L (reference range: 0-14 ng/L). The patient was admitted to the pediatric intensive care unit with a differential diagnosis of acute myocardial infarction or acute myocardial injury related to cardiomyopathy and commenced on an ACE (angiotensin-converting enzyme) inhibitor. Computed tomography (CT) of the coronary arteries was performed, which showed normal coronary arteries and cardiac anatomy.

The patient was discharged on day 5 and continues to follow up in the pediatric cardiology clinic. He was commenced on a beta blocker at one-month follow-up when he was asymptomatic.

## Introduction

Duchenne muscular dystrophy (DMD) is an X-linked disease that usually results from a dystrophin gene abnormality. Absence of dystrophin protein in skeletal and cardiac tissue results in contractile protein degradation, fibrosis, and apoptosis [1]. It affects one in 5,000 live male births, with approximately 20,000 new cases worldwide yearly [2,3].

Advancements in respiratory care have improved life expectancy to the late 20s, with cardiomyopathy emerging as the most prevalent cause of death [1-4].

Elevated liver transaminases and creatine kinase often trigger a referral to neurologists, and current standards advise performing genetic testing for the DMD gene first before muscle biopsy [3,5].

Children usually present with progressive neurological deficit characterized by gait disturbances, speech problems, and proximal muscle weakness. Weakness starts in the proximal lower limbs and trunk, later involving the upper limbs and distal muscles [6]. The majority are wheelchair-dependent before their teens [7].

We present a case of a 12-year-old boy who presented to our Emergency Department (ED) with no previous records of baseline investigations at our hospital and a chief complaint of central crushing chest pain and electrocardiogram (ECG) changes suggestive of myocardial ischemia.

## Case presentation

**Figure 1:**
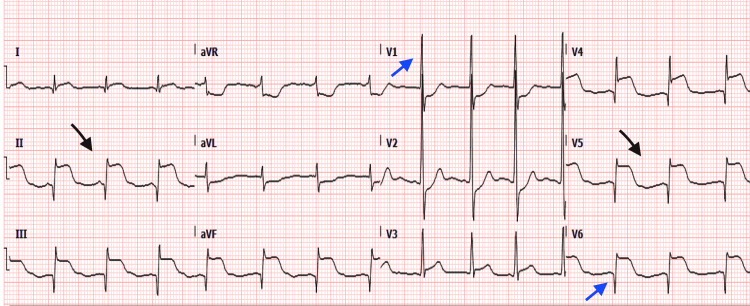
A 12-lead ECG showing ST-segment elevation in the inferior and lateral leads of more than 2 mm (shown with black arrows) with corresponding reciprocal changes. Also visible are signs of right ventricular hypertrophy, i.e. right axis deviation, a dominant R wave in lead V1, and dominant S waves in leads V5 and V6 (shown with blue arrows).

A 12-year-old boy presented to our ED complaining of crushing central chest pain, which started 12 hours prior, radiating to his back and jaw. A 12-lead ECG at first medical contact showed inferolateral ST-segment elevation with reciprocal changes (Figure 1), He was loaded with aspirin and administered intravenous morphine.

The patient had a medical background of DMD and was receiving oral prednisolone every other day. He had no known cardiac anomalies, and an echocardiogram performed in 2018 showed a structurally normal heart. There was no history of smoking or recreational drug use. Due to progressive neuromuscular weakness, he became wheelchair-dependent by the age of 10 years.

Clinical examination revealed an overweight male (BMI 24.8 kg/m2, 95th percentile). His vital signs were normal except a resting tachycardia of 103 beats per minute. The case was discussed with the interventional cardiologist as well as the pediatric cardiologist, the latter based in a network hospital in the same city. Initial cardiac biomarkers were significantly elevated, with troponin-T result recorded at 7,065 ng/L (reference range: 0-14 ng/L) using the Elecsys® troponin T-high sensitive assay (Roche Diagnostics, Basel, Switzerland). The patient was admitted to the pediatric intensive care unit with a differential diagnosis of acute myocardial infarction or acute myocardial injury related to cardiomyopathy. Inflammatory markers and an infection screen were also performed, which were negative for any extrinsic infectious etiology for this presentation.

Based on the presentation to ED with crushing chest pain, the ECG changes noted, and the elevated cardiac biomarkers, an initial working diagnosis of acute myocardial infarction was made.

Another differential diagnosis was cardiomyopathy-related acute myocardial injury. Cardiomyopathy is more commonly described in DMD. In this case, the cardiomyopathy may have been largely asymptomatic prior to presentation. On attendance in ER, the clinical features were not consistent with heart failure, and a structurally normal heart was seen on echocardiography; however, it should be noted the absence of typical symptoms of heart failure such as dyspnea on exertion and limited exercise tolerance are common in DMD. Furthermore, in early stages, due to restricted left ventricular (LV) enlargement, the echocardiogram may appear normal [3].

**Figure 2:**
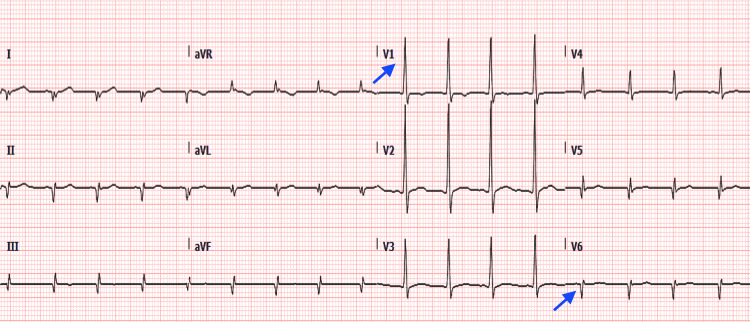
A 12-lead ECG on day 5 showed the ST elevation visible earlier had resolved but signs of right ventricular hypertrophy persisted, i.e. right axis deviation, a dominant R wave in lead V1, and dominant S waves in leads V5 and V6 (shown with blue arrow).

A 12-lead ECG in the ED showed a sinus rhythm and ST-segment elevation in the inferior and lateral leads of more than 2 mm with corresponding reciprocal changes. Signs of right ventricular hypertrophy (RVH), i.e., right axis deviation, dominant R wave lead V1, and dominant S waves in leads V5 and V6 (Figure 1) were also demonstrated. A 12-lead ECG on day 5 showed the ST-segment elevation had resolved but signs of RVH persisted (Figure 2). We did not have access to a baseline ECG to compare the current attendance’s ECG changes with.

The patient was admitted to the pediatric intensive care unit for monitoring and medical management for 5 days. He was commenced on captopril, and his usual treatment plan with steroids was continued.

An echocardiogram was performed on admission, which showed depressed LV systolic function with an ejection fraction (EF) of 45%, mild insufficiency of the aortic valve, and no structural anomalies. Cardiac CT angiography of the coronaries was performed the next day, which demonstrated normal cardiac anatomy and normal coronary arteries. Unfortunately, our patient did not undergo cardiac magnetic resonance imaging (CMR) on admission.

Serial troponins were collected on a daily basis, which showed a decreasing trend with a troponin level of 1,271 ng/L recorded on the day of discharge. A repeat echocardiogram was performed before discharge, which showed an improved EF of 58% and normal ventricular systolic function.

At eight-week follow-up, a repeat echocardiogram was reported showing mildly depressed LV systolic function with an EF of 49% and mild insufficiency of the aortic valve. Troponin-T assay was now recorded at a level of 74.8 ng/L. The patient was commenced on bisoprolol and continues to follow up in the clinic.

## Discussion

Cardiac disease in DMD has been described preclinically as young as in toddlers. By the age of 18, the vast majority of DMD patients have cardiac disease, with the commonest being cardiomyopathy. Dilated cardiomyopathy is more common followed by hypertrophic cardiomyopathy. Symptoms are often absent due to restricted physical activity [1,8].

Arrhythmias such as atrial tachycardias, ventricular tachycardia, and ventricular fibrillation have been reported in advanced cardiomyopathy. Sinus tachycardia is the most common arrhythmia observed [3,9]. Heart blocks are also reported, although these are less common. Severe LV dysfunction is a risk factor for arrhythmias, and late gadolinium enhancement (LGE), a marker for myocardial fibrosis, seen on cardiac CMR, is also associated with a higher risk of arrhythmias [1,3].

Abnormal ECG findings are found in 70% of cases, including sinus tachycardia, short PR interval, dominant R wave in lead V1, deep Q waves in the inferolateral leads, RVH, aberrant conduction, and a prolonged QT interval [10].

Cardiac biomarkers can be chronically elevated due to their skeletal muscle origin, with cardiac troponin I (cTnI) being more specific than cardiac troponin T (cTnT) possibly because it is not expressed in human skeletal muscle and is strongly associated with cardiovascular disease [11,12]. Furthermore, cut-off levels of troponin for the diagnosis of acute coronary syndrome are not validated in children and in children with DMD. In a case series by Hor et al., cTnI levels ranged from 31 ng/ml to 62 ng/ml (reference range: <0.03 ng/ml) [13]. Similarly, ECG criteria for diagnosing acute myocardial infarction are not well-defined or validated in children. Towbin et al. suggested criteria based on ST-segment and Q wave changes [14].

Echocardiography has been used for the assessment of cardiac wall motion and EF due to it is availability and low cost. It has limitations in being operator-dependent. CT coronary angiography might be a useful tool for assessment if CMR is not readily available. CMR is recommended for screening of disease progression showing hallmark changes in the posterobasal and basal inferiolateral areas of the left ventricle [15]. LGE in the subendocardium suggests ischemic injury, whereas subepicardial localization favors myocarditis or an infiltrative disorder [16]. In DMD, it is recommended CMR is conducted annually after the age of 10 years.

Hor et al. reported eight DMD pediatric cases who presented with chest complaints, ST-segment elevation, and elevated troponin levels. In all eight patients, normal coronary perfusion was confirmed by CT angiography or cardiac catheterization, whereas on CMR, abnormal systolic function was demonstrated [13]. They attributed this to cardiomyopathy disease progression, which leads to episodic myocardial injury. Cinteza et al. described the use of pulsed steroid therapy as a treatment of acute myocardial injury in a symptomatic child with DMD with favorable results [15]. Another study described an asymptomatic 12-year-old child with DMD who was diagnosed with acute myocardial injury by ST-segment elevation and elevated cardiac biomarkers. They compared apoptosis and necrosis as mechanisms of cardiac cell death [17]. There are multiple case reports describing myocardial infarction in DMD children aged 10-13 years [18-21]. One study described myocardial infarction in a 10-year-old DMD child following physical exertion and another in 13-year-old DMD patients who experienced acute chest pain and had ST-segment elevation on their ECG and high troponin levels [20,21].

The principle of pharmacological treatment in DMD is to delay onset of heart failure. Early steroid therapy has a beneficial impact on lung function, LV function, and skeletal muscle. Similarly, use of angiotensin-converting enzyme (ACE) inhibitors has been proven to delay the onset and progression of LV dysfunction [22].

Once LV dysfunction is established, corticosteroids remain beneficial and are shown to delay establishment of myocardial fibrosis on CMR [23]. ACE inhibitors are recommended first-line therapy proven to reduce mortality and hospital admission in DMD patients with heart failure [24].

Beta blockers have also been shown to be beneficial in DMD as an anti-arhythmic, improve EF, and reduce detrimental ventricular remodelling [25]. Aldosterone antagonists such as eplerenone have also had a positive impact when started at a younger age, in addition to ACE inhibitors and beta blockers [8,26].

Some anti-arrhythmic drugs can increase skeletal muscle weakness, otherwise their use in DMD patients is similar to non-DMD patients [27].

In patients with end-stage heart failure, use of mechanical cardiac support devices is also a feasible option. Similarly, non-invasive ventilation is a treatment option for restrictive ventilatory defects resulting from progressive respiratory muscle weakness [28].

DMD patients can attend ED with many acute problems, with respiratory complaints perhaps being the commonest. Dyspnea is often mild compared to the severity of disease. Undifferentiated chest pain from cardiac and non-cardiac causes are both likely. Esophagitis and musculoskeletal chest pain, with the latter from coughing, have been reported. Constipation is also common but look for other high-risk conditions first such as steroid-related ulcer disease. Renal colic is another differential diagnosis when presenting with abdominal pain. They are also prone to back pain from osteoporotic vertebral fractures. Fatigue is also reported possibly attributed to nocturnal hypoventilation, mood disturbances, and advanced cardiomyopathy. Have a low threshold to discuss the care of DMD patients with their parent team, often the neurologists [28].

It is worth noting that 5% of all myocardial infarctions do not have obstructed coronary arteries, a condition termed “myocardial infarction with normal coronary arteries” (MINOCA). In these cases, sepsis, pulmonary embolism, substance misuse, myocarditis, or oxygen supply-demand mismatch resulting in type 2 myocardial infarction (e.g. from anemia or thyrotoxicosis) should be excluded [16,29]. In children, MINOCA from myocarditis has been reported to be associated with sudden cardiac death in 5% of cases [29]. Pediatric Kounis syndrome, a hypersensitivity coronary disorder involving mast cells and other inflammatory mediators, has also been reported as an etiology of MINOCA [30].

## Conclusions

In conclusion, our patient with chest pain and ECG changes suggestive of myocardial ischemia was objectively evaluated for coronary artery disease. It is plausible our patient had episodic myocardial injury due to myocardial disease progression, with the latter being a common cause of morbidity and mortality in DMD patients.

DMD patients can present to ED with a variety of complaints that are often respiratory or cardiac in nature. It is also worth remembering that vertebral compression fractures and other fragility fractures are common in children with DMD due to osteoporosis and steroid use. Progressive muscle weakness eventually leads to complete loss of mobility. Respiratory muscle weakness may result in mucus plugging, atelectasis, pneumonia, and respiratory failure.

Frontline healthcare practitioners should be aware of the high prevalence of cardiomyopathy in these patients, which increases with age. Such patients should have an ECG performed, which should be compared to a baseline ECG if possible. As a rule, be vigilant and discuss these cases early on with their parent team. Cardiac biomarkers can be chronically elevated in DMD. However, a high level of suspicion coupled with an echocardiogram can assist in the diagnosis of acute myocardial injury in these cases. Where available, CMR assists in the evaluation of patients with cardiomyopathy-related myocardial injury, with characteristic LGE seen.
